# Receptor Tyrosine Kinases and Inhibitors in Lung Cancer

**DOI:** 10.1100/tsw.2004.117

**Published:** 2004-08-06

**Authors:** Evan Pisick, Simha Jagadeesh, Ravi Salgia

**Affiliations:** ^1^ Tufts-New England Medical Center, Tufts University School of Medicine, Boston MA, USA; ^2^ University of Chicago, Section of Hematology and Oncology, Pritzker Medical School, Chicago, IL, USA

**Keywords:** RTK, EGFR, c-Kit, c-Met, VEGF, IGF, Eph, ephrins

## Abstract

Lung cancer is a deadly disease with high mortality and morbidity. Most cases of lung cancer are due to non-small cell carcinoma, with 16% of cases being small cell carcinoma. The biology at a cellular level is of interest at many levels. Knowing cellular pathways helps to further enhance our knowledge of how lung cancer cells survive, proliferate, and metastasize. The receptor tyrosine kinases (RTKs) located at the cellular membrane are becoming of great interest as sites for targeted therapies for lung cancers. This review will discuss the RTKs that are involved in lung cancers and the newer therapies that are being tested. We will specifically discuss receptors such as epidermal growth factor receptor, c-Kit receptor, VEGF receptor, c-Met receptor, insulin growth factor receptor, and Eph receptor. The inhibitors against the specific RTKs are in various preclinical and clinical trials, and this will be detailed.

## INTRODUCTION

In the year 2004, approximately 173,770 new cases of lung cancer will be diagnosed. Of this number, 16% are small cell lung cancer (SCLC) with the rest being non-small cell lung cancer (NSCLC). Both diseases are associated with exposure to smoking, asbestos, and potential other aerosolized carcinogens. Currently these diseases are treated with surgery, thoracic radiotherapy, and cytotoxic chemotherapies. Only recently have newer therapies been developed that have specific targets within or on the cell membrane[1].

Lung cancer cells, like normal cells, express receptor tyrosine kinases (RTKs). The difference is that these receptors may have altered function or numbers. They may be overexpressed or mutated leading to increased activation. Currently, more than 60 RTKs have been identified and all share similar structure[2]. RTKs contain an N-terminal extracellular domain for ligand binding, a single alpha-helix transmembrane domain, and a C-terminal domain with tyrosine kinase activity located in the cellular cytoplasm[2]. These receptors are the beginning of several different cellular pathways involved in all aspects of cellular function including proliferation, apoptosis, and motility.

## RECEPTOR TYROSINE KINASES

RTKs have a conserved structure among the vast majority. The extracellular domain is a site for ligand binding[3]. It is this binding that leads to activation of the intracellular catalytic domain and potentiation of tyrosine kinase activity[4]. The tyrosine kinase then autophosphorylates tyrosine residues, which act as docking sites for cytoplasmic adapter molecules and thus activate signal pathways that result in a myriad of cellular responses.

Several RTKs have been described in lung cancers as well as their functions. These receptors are proto-oncogenes and play an important role in transformation, proliferation, motility, and survival of cancerous cells. RTKs can be overexpressed or have abnormally increased function due to mutation[5,6]. Several RTKs will be described in terms of structure, expression, and role in biologic and biochemical functions within lung cancer. RTKs are also becoming targets for therapy for patients with tumors that overexpress these receptors[7,8,9,10]. Several methods of inhibition are being developed, including small molecules that inhibit tyrosine kinase activity and monoclonal antibodies that interfere with ligand binding to the RTKs[11], antisense methodology[12], as well as ribozyme targeting RTKs[13]. In this review, RTKs to be discussed will include the HER family including the epidermal growth factor receptor (EGFR), insulin-like growth factor receptor (IGFR), vascular endothelial growth factor receptor (VEGFR), c-Kit, c-Met, and the ephrin family of RTKs. All of these appear to have an important role in lung cancer and thus serve as attractive targets for inhibition.

### Epidermal Growth Factor Receptor (EGFR)

EGFR, also known as HER 1 or c-erbB-1, is part of a family of RTKs that include the c-ErbB-2/HER2/neu, c-erbB-3/HER3, and c-erbB-4/HER4 receptor tyrosine kinases[14]. There is also a mutant type EGFR receptor, EGFRvIII, which lacks residues 6-276 in the extracellular binding domain. This altered RTK results in ligand-independent activation of the receptor in a constitutive form[15]. EGFR is a 170-kDa glycoprotein that contains two cysteine-rich repeats in its extracellular domain, a single alpha-helix transmembrane domain, a catalytic domain, and a C-terminus with multiple tyrosine residues that have the ability to bind to SH2 domains located on various adapter molecules[16]. This receptor is activated by several different ligands that include epidermal growth factor (EGF), transforming factor-β, betacellulin, amphiregulin, heparin-binding EGF, and epiregulin.

The RTKs in the c-erbB family have a high degree of homology in the tyrosine kinase domain, but their extracellular domains are less conserved. This lack of homology in the extracellular domain is the reason for each receptor being able to bind to different ligands. c-erbB-2 is the only member of this family for which no ligand has yet been identified. It has also been shown that the c-erbB-3 RTK lacks intrinsic tyrosine kinase activity[17]. On ligand binding to the receptor, homo- and heterodimerization occurs. Heterodimers are important, as c-erbB-3 homodimers are inactive due to lack of tyrosine kinase activity. HER2/neu and c-erbB-3 RTKs can only be activated by forming heterodimers with any of the three other RTKs.

Once dimerization occurs, autophosphorylation of the RTKs occurs as the catalytic domains are activated and brought into close proximity of another receptor. After this event occurs, several pathways are activated that result in cell proliferation, differentiation, migration, adhesion, and protection from apoptosis. Several intermediaries have been elucidated through extensive research. Pathways that are induced include Ras-Raf-MEK-mitogen–activated protein kinase (MAPK)[18], phosphotidylinostitol 3-kinase (PI3-K)-Akt[19], PLC-γ[20], Src[20], PAK-JNKK-[20], and stress-activated proteins.

Motility, migration, and invasion by cancerous cells are tightly regulated by RTKs[2]. Motility is dependent on the actin cytoskeleton that interacts with the extracellular environment at focal adhesions. In this specialized portion of the cell, several cytoskeletal proteins are located and include PI3-K, Rho family members (Rho, Rac, Cdc42), and focal adhesion proteins (paxillin, p125FAK, and talin). It has been shown that breast cancer cells that overexpress EGFR have increased motility when exposed to EGF[21]. This phenomenon has also been described in SKBR3 cells overexpressing EGFR as well. Activation of EGFR by its ligands can also lead to alteration of several cellular pathways through several different intermediaries. Reactive oxygen species (ROS) including $O2^{-}$, OH, NO, H2O2 play an important role in signal transduction, motility, proliferation, and gene expression. Activation of EGFR can lead to the generation of ROS, which can then lead to the phosphorylation of EGFR. This process can lead to altered downstream phosphorylation of other molecules as well as decrease the turnover time of EGFR.

EGFR's role in oncogenesis was first described in NIH3T3 cells by overexpression, stimulation with EGF, and tumor formation in nude mice. Since then, a variety of solid tumors have been shown to overexpress EGFR[22,23]. These tumors include lung, breast, prostate, ovary, stomach, colon, larynx, and glioblastomas[24]. It has also been shown that overexpression of EGFR correlates with a poorer prognosis and stage of disease in patients with NSCLC. EGFR, HER2/neu, and c-erbB-3 are present and overexpressed in 40–80, 18–60, and 25–85% of lung cancers, respectively.

Molecules that inhibit EGFR activity are being developed and tested[25]. They include small molecules as well as monoclonal antibodies. A monoclonal antibody that binds EGFR in a similar manner to EGF has been developed[26]. IMC-C225 is a human/murine chimeric antibody. The action of this antibody may be twofold. It inhibits EGFR signaling by blocking the receptor and also may play a role in recruiting immune cells leading to antibody-dependent cell-mediated cytotoxicity. In human NSCLC xenograft models, IMC-225 has been shown to enhance the cytotoxic effects of chemotherapeutics and radiation[27]. This antibody has been extensively studied in patients with colon cancer and head and neck cancers, and is now being tested in patients with NSCLC. Several trials are ongoing using IMC-225 in combination with chemotherapeutics in patients with either chemotherapy-naïve or -refractory NSCLC. Patients in these studies must have NSCLC that expresses EGFR.

Inhibitors block the kinase activity of RTKs by binding either reversibly or irreversibly to the catalytic domain in the cytoplasm. This would prevent autophosphorylation of the activated RTKs and thus inhibit their downstream signaling pathways. Several different inhibitors have been produced and are at various stages of clinical testing in patients with cancer.

The first is Iressa (ZD1839), which is an orally available quinazoline-based agent that binds reversibly to the EGFR tyrosine kinase rendering it inactive. This inhibitor has been shown to induce cell-cycle arrest and apoptosis by decreasing the activity of the PI3-K/Akt pathway. To fully demonstrate this, Bianco et al. conducted experiments using the EGFR expressing A431 NSCLC and MDA-468 breast cancer cell lines. They demonstrated that PTEN-null MDA-486 cells were relatively resistant to Iressa at high concentrations, while A431 cells (wild-type PTEN) were sensitive to this small molecule at low levels[22].

Iressa-treated A431 cells led to reduced association between EGFR, HER3, and the p85$\alpha$ subunit of PI3-K, which in turn led to decreased phosphorylation of Akt. Although a similar decrease in association between EGFR, HER3, and PI3-K was seen in MDA-468 cells treated with Iressa, the levels of phosphorylated Akt remained unchanged. Further studies showed that if PI3-K was inhibited, then phosphorylation of Akt was decreased in both the MDA-468 and A431 cell lines, which demonstrates that Akt activation in MDA-468 is independent of EGFR signaling[22].

One difference between these two cell lines was the presence of PTEN. MDA-468 cells were transfected with full-length PTEN, which resulted in an increased sensitivity and rate of apoptosis when exposed to Iressa; MDA-468 cells transfected with an empty vector continued to be resistant to the effects of Iressa. The PTEN expressing A431 and MDA-468/PTEN cells both exhibited decreased phosphorylation of Akt when exposed to Iressa[22].

Iressa is the most extensively tested EGFR inhibitor tested in patients with advanced NSCLC[28,29]. Two larger phase II trials were conducted in NSCLC patients who previously failed at least one platinum-based chemotherapy regimen. The first study was the Iressa Dose Evaluation in Advanced Lung Cancer (IDEAL 1) and was conducted in Europe, Japan, Australia, and New Zealand. This study evaluated both the efficacy and tolerability of two different doses of Iressa, 250 and 500 mg/day. The trial included 210 previously treated patients with refractory or recurrent stage III/IV NSCLC in a randomized double-blinded fashion. Results revealed equal efficacy between the two doses. Objective tumor response rates were 18.4 and 19%; symptom improvement rates were 40.3 and 37%; median progression-free survival times were 2.7 and 2.8 months; and median survival times were 7.6 and 8 months, respectively. The results of this trial led to the Japanese FDA approval for use of this drug in patients with advanced refractory or recurrent NSCLC[30,31].

A second trial in the U.S. was conducted in a similar patient population. This study was called the IDEAL 2 trial. This study was designed to evaluate symptomatic and radiographic response among patients with stage IIIB/IV NSCLC. Patients had to have previously received a platinum-based chemotherapy and now have recurrent or refractory disease. Once again two different doses, 250 and 500 mg/day, were compared. In patients receiving 250 or 500 mg/day, symptomatic improvement occurred in 43 and 35% and partial radiographic response in 12 and 9%, respectively. Of the patients with radiographic responses, 96% had a symptomatic improvement. The overall 1-year survival rate was 25%[32,33].

Iressa has also been tried in patients with chemotherapy-naïve NSCLC in combination with platinum-based regimens. The first trial, Iressa NSCLC Trial Assessing Combination Treatment 1 (INTACT 1), accrued 1093 patients with stage III/IV disease that had never before received chemotherapy. All patients received six cycles of gemcitabine and cisplatin and were randomized to either placebo, or Iressa at 250 or 500 mg/day. The placebo or Iressa were taken until disease progression was noted. Overall survival, progression-free survival, and time to worsening of symptoms were similar in all three treatment arms[34]**.** A second trial, INTACT II, randomized 1037 patients with chemotherapy-naïve stage III/IV NSCLC to receive six cycles of carboplatin and paclitaxel with either placebo, or Iressa at 250 or 500 mg/day. This study had the same results as the INTACT 1 trial, showing no difference in overall survival, progression-free survival, and time to worsening of symptoms between the three groups[35].

While the trials, overall, showed only a modest benefit from Iressa on NSCLC, 10% of patients do show a dramatic clinical response to the drug. Recently, two separate studies[36,37] revealed a relationship between activating mutations of the EGFR receptor and clinical response to therapy. In one study[36], somatic mutations in the tyrosine kinase domain were demonstrated in eight out of nine patients who responded to therapy, as compared to no mutations in seven of the patients with no response. *In vitro*, EGFR mutants also showed increased sensitivity of the tyrosine kinase domain to EGFR and increased inhibition by Iressa. In the other study[37], RTKs were sequenced in NSCLC vs. matched normal tissue and mutations were found in 15 of 58 tumors from Japan and 1 of 51 tumors in the U.S. Response to Iressa, demonstrated by tumor regression, is more common among the Japanese patients with NSCLC. Additionally, mutations were also found in lung cancer samples from U.S. patients who responded to Iressa. *In vitro*, a mutation was also found in a cell line that was hypersensitive to growth inhibition by Iressa, but not in cell lines that were insensitive to the drug. These overall results suggest that the Iressa is more effective in tumors bearing activating genetic mutations of the EFGR receptor, rendering the tumor biologically dependent on EFGR. This phenomenon may also be present in the context of other RTKs, possibly making it possible to predict the response to the inhibitors of these RTKs.

A second inhibitor, Tarveca (OSI-774), is being examined in patients with NSCLC[38]. This agent is also an orally available reversible quinazoline-based agent with tyrosine kinase–inhibiting ability. This RTK inhibitor also causes cell-cycle arrest and apoptosis[38]. Three phase II clinical trials were conducted in patients with previously treated NSCLC, ovarian, and head and neck cancers[39,40]. Patients with chemoresistant NSCLC that were expressing EGFR were studied[41]. One of 57 (1.8%) patients had a complete response, 8 of 57 (14%) achieved a partial response, and 15 of 57 (26%) had stabilization of their disease. Median survival was 37 weeks with a 1-year survival rate of 48%. Currently, two large phase III trials are being conducted in which patients are receiving Tarveca with either a combination of cisplatin and gemcitabine or carboplatin and paclitaxel as first line therapy for patients with NSCLC; enrollment for these trials has been completed. Other trials are also being started with the use of Tarveca as monotherapy or in combination with single agent chemotherapies as second-line treatments. In a recently published[42] randomized placebo-controlled trial of Erlotinib in patients with advanced NSCLC following failure of first- or second-line chemotherapy, overall response to Erlotinib was 8.9% for a median duration of 34.2 weeks. Statistically significant and clinically relevant differences were observed for overall survival and progression-free survival. This was the first trial to confirm that an EGFR inhibitor can prolong survival in NSCLC, after first- and second-line chemotherapy.

There are several other RTK inhibitors now entering preclinical and clinical development. These include EKB-569, PKI-166, GW572016, and CI-1033[43]. EKB-569 is a 3-cyanoquinoline that irreversibly inhibits both HER1 and HER2 RTKs in cells overexpressing these receptors. PKI-166 is a pyrrolopyrimidine that reversibly inhibits the HER1 and HER2 RTKs. This agent in entering phase I trials. GW572016 is a 6-thiazolyquinizoline that also inhibits the HER1 and HER2 RTKs. CI-1033 is an irreversible inhibitor also entering phase I trials.

## c-Kit c-Kit is a type III RTK similar to c-Fms, Flt3, and platelet-derived growth factor receptor (PDGFR). It is a 145-kDa protein containing five extracellular immunoglobulin-like domains, a single transmembrane domain, and a cytoplasmic domain with a split kinase domain and a hydrophilic kinase insert sequence[44,45,46]. The ligand for c-Kit is the stem cell factor (SCF), also known as steel factor. This RTK is expressed in numerous normal tissues, but is aberrantly expressed in 30–40% of SCLC tumors[47] and cell lines and 40% of NSCLC tumors. Other tumors that overexpress c-Kit include GI stromal tumors (GIST), breast, cervical, ovarian, and melanomas. This receptor may be activated in an autocrine and/or paracrine fashion by its ligand, be cross-activated by other kinases, or have activating mutations[48].

Cell growth and motility are but two functions that are affected by c-Kit and its ligand SCF. Abnormal cell growth is seen in SCLC and is caused by c-Kit/SCF acting in both autocrine and paracrine fashions. c-Kit/SCF stimulation of the cell leads to activation of various cellular pathways. In H526 cells, a SCLC cell line, addition of SCF to culture medium results in activation of both MAP kinase and Akt[49]. In Mo7e cells, stimulation with SCF leads to a rapid increase in reactive oxygen species and thus increases phosphorylation of multiple proteins including STAT5, a molecule involved in viability signaling[50]. This affect is negated by exposing the Mo7e cells to antioxidants such as pyrrolidine dithocarbonate (PDTC), N-acetyl cysteine, and 2-mercaptoethanol. Focal adhesion proteins such as paxillin and p125FAK are phosphorylated in response to c-Kit stimulation in hematopoietic cells. It has been shown that cell growth is inhibited when a kinase defective c-Kit is introduced into a c-Kit/SCF expressing cell line[51]. The tyrosine kinase inhibitor STI571 (formerly known as CGP57148B, currently as Gleevec, Novartis Pharmaceuticals) is a competitive inhibitor developed against the Abl protein kinase. This agent was first used to inhibit the tyrosine kinase of the BCR/ABL oncogene in patients with chronic myelogenous leukemia (CML). This kinase inhibitor is now being shown to be nonselective and activity against c-Kit and PDGFR has been reported.

Since 30–40% of SCLC express c-Kit, SCLC cells have been tested against STI571 (Gleevec, Novartis). Growth of some SCLC cell lines has been shown to be inhibited by this agent[52]. SCF-induced activation of both MAPK and Akt were inhibited by pretreatment of cells with STI571. The drug works in a cytostatic manner and apoptosis does not occur. This is partially due to the fact that both MAPK and Akt are weakly activated when these cells are incubated with IGF-I[49]. This result suggests that multiple pathways are involved in the growth of SCLC cell lines. Although this drug has shown effect *in vitro*, it has not yet shown any clinical effect in patients with SCLC[53].

In a limited phase II trial of STI-571 in SCLC done by Johnson et al.[54], there was no observed antitumor activity among 19 patients. Among the 19 patients enrolled in the study, 9 had previously untreated extensive stage disease and 10 had sensitive relapse. The median time to progression was 0.8 months (range, 0.6–1.3 months) and 1.2 months (range, 0.2–4.1 months) in the previously untreated and sensitive-relapse groups, respectively. Tumor analysis by immunohistochemistry showed that only 4 patients showed the expression of the c-Kit receptor.

Several other tyrosine kinase inhibitors have been experimented with in SCLC cell lines and include SU5416, SU6597, SU SU5614, SU6668, SU6663, and SU6561[55]. These agents were shown to have to inhibit the c-Kit RTK, but had varying potencies toward other tyrosine kinases, including poor potency to the IGF-I kinase. SU6597 and SU5416 were the most active in H526 cells and subsequently showed activity in multiple SCLC cell lines. Apoptosis was induced in these cells at higher concentrations of these two inhibitors, but although apoptosis was induced, this effect was negated when IGF-I was present in the culture medium. This result suggests that multiple pathways are involved in the growth of SCLC cells.

## Vascular Endothelial Growth Factor Receptor (VEGFR)

VEGFR is a family of receptors with multiple members. The family includes VEGFR-1 (Flt-1), VEGFR-2 (Flk-1/KDR), VEGFR-3 (Flt-4), and neutropilin-1. These RTKs consist of an extracellular domain that contains seven immunoglobulin-like regions, a transmembrane region, and an intracellular domain that contains a tyrosine kinase region[56]. These receptors have multiple ligands that activate intracellular pathways. These ligands include VEGF-A, VEGF-B, VEGF-C, and VEGF-D. Overexpression of VEGFs is associated with angiogenesis in both primary and metastatic cancers[57].

VEGF activates many diverse functions within the cell. Activation of the RTKs in the VEGFR family leads to promotion of endothelial cell mitogenesis, promotion of endothelial cell survival, chemotaxis, increased expression of proteolytic enzymes involved in stromal degradation, increased vascular permeability, inhibition of maturation of antigen-presenting dendritic cells, and vasodilatation[58]. Different RTKs in this family are linked to one or more of the preceding cellular functions. VEGFR-1 is thought to be important in migration[59,60], while VEGFR-2 may be important in the induction of vascular permeability and cellular proliferation, mitogenesis, and chemotaxis[61]. Activation of VEGFR-3 has been shown to be involved in regulation of lymphatic angiogenesis[62]. Neutropilin-1 enhances the activation of VEGFR-2 by enhancing its binding to ligand[63].

There are several different ligands that are involved in activation of cellular pathways linked to the VEGFR family of RTKs, but each receptor only interacts with a few of the previously named ligands. VEGF-A is a 34- to 50-kd homodimeric glycoprotein produced by both malignant and normal cells[58]. The expression of this molecule is regulated by differentiation and transformation, hypoxia, glucose concentration, and pH[56]. There are also several growth factors, cytokines, and hormones that can upregulate VEGF-A expression[64]. Hypoxia-inducible factor-1 can bind the promoter gene of this protein and activate transcription in response to oxygen deprivation. In most human tumors, VEGF-A expression is increased[65]. Increased levels are associated with a greater microvascular density and a poorer prognosis.

VEGF-A can undergo alternative splicing and produce at least five different variants. The most predominant and biologically active isoform is VEGF165[64]. VEGF-A interacts with three members of this RTK family and includes VEGFR-1, VEGFR-2, and neutropilin-1[66,67]. When VEGFR-2 and neutropilin are coexpressed, binding of VEGF165 to VEGFR-2 is enhanced by neutropilin-1. VEGF-A binding to its receptor activates several different pathways. Antiapoptotic proteins such as bcl-2 and A1 are induced by VEGF-A binding to its receptors[68]. There is also activation of the phosphatidylinositol 3-kinase/Akt pathways, stimulation of nitrous oxide and PGI2, and increased tyrosine phosphorylation of p125FAK[69].

VEGF165 does not interact with the other RTKs of this family, but VEGF-C and VEGF-D bind to VEGFR-3[62]. This binding controls growth and maintenance of lymphatic vessels. VEGF-B interacts with VEGFR-1 and may play a role in the cardiovascular system[70,71,72].

As with other RTKs, several small molecules targeted towards the tyrosine kinase region of the receptor have been developed. The first two include SU5416 (Pharmacia, Sugen) and SU6668 (Pharmacia, Sugen), both of which have been shown to inhibit the growth of SCLC cell lines. The former is a VEGFR-2 tyrosine kinase inhibitor, while the latter is less specific and inhibits the tyrosine kinase of VEGFR-2, basic fibroblast growth factor receptor, and PDGFR. These two agents have been studied *in vivo* in a murine colon cancer liver metastatic model; there was decreased liver metastasis liver weight[73]. It is suggested that inhibition of VEGF activity indirectly leads to tumor cell death as histopathology of metastases revealed necrotic areas. SU5416 has no direct effect on colon cancer cells *in vitro*, so it is hypothesized that it is disruption of the tumor vasculature that leads to cellular apoptosis. A follow-up study in mice revealed a survival improvement in mice receiving SU6668 over those receiving placebo[74]. A third molecule, PTK787/ZK222584 (Novartis) inhibits the tyrosine kinase of both VEGFR-1 and VEGFR-2 and is currently finishing preclinical trials[75].

In a study by Kuenen et al.[76], to investigate the feasibility and pharmacokinetics of SU5416 in combination with gemcitabine and cisplatin, a high incidence of thromboembolic events were observed. Among 19 patients involved, 8 developed 9 thromboembolic events (including 3 transient ischemic attacks, 2 cerebrovascular accidents, and 4 deep venous thromboses). Analysis of variables of the coagulation cascade and of vessel wall activation was performed in 3 patients and showed significant increases in thrombin generation and endothelial cell perturbation in a treatment cycle-dependent manner. The severe toxicities of this regimen discouraged any further investigation into its efficacy.

Monoclonal antibodies have also been developed against VEGF. Genentech (South San Francisco, CA) has developed a humanized monoclonal antibody that recognizes all known isoforms of VEGF (rhuMAb VEGF). A phase II trial is being conducted involving multiple tumors, including NSCLC, as both a single agent and in combination trials[66]. Sledge[77], using rhuMAb VEGF as a single agent, were able to induce remission and prolong stabilization of disease in patients with heavily pretreated metastatic breast cancer. The Eastern cooperative Oncology Group is currently performing a phase III trial in patients with NSCLC using rhuMAb VEGF.

Monoclonal antibodies are also being developed against the VEGFRs as well. DC101 is being combined with low-dose vinblastine in preclinical models in mice with neuroblastoma. These studies have shown sustained remissions of large tumors.

## c-Met c-Met belongs to a family of heterodimeric tyrosine kinases which includes Ron, Ryk, Sea, and Sex. This is a protein with an extracellular alpha chain and beta-subunit that contains the ligand binding site, a single transmembrane domain, and an intracellular tyrosine kinase catalytic domain. A single precursor weighing 170 kDa is cleaved and glycosylated to obtain both of the subunits. c-Met is activated by its ligand hepatocyte growth factor (HGF), also known as scatter factor, which causes phosphorylation at multiple sites, with the major site located at Y1230/1234/1235. Coexpression of c-Met and HGF has oncogenic potential and has shown to transform NIH3T3 cells.

This RTK is overexpressed in tumors such as lung (both SCLC and NSCLC), thyroid, and pancreatic. c-Met is mutated in other carcinomas such as the hereditary and sporadic forms of papillary renal cell carcinomas. Expression of c-Met has been shown to correlate with higher pathologic tumor stage and worse prognosis. NSCLC and SCLC cell lines have been shown to express c-Met, as well as 72% of NSCLC adenocarcinomas and 38.5% of NSCLC squamous carcinomas. We have recently shown there are specific mutations of c-Met, especially in the semaphorin domain and juxtamembrane domain[78].

Stimulation of c-Met by its ligand HGF causes increased cell motility and works in a paracrine fashion[79,80]. HGF-induced activation of c-Met leads to activation of several downstream molecules, thus enhancing cell movement[81]. PI3-K and p21GTPases Ras, Rac, and Rho all appear to be important molecules in HGF-stimulated cell migration. In A549 NSCLC cells, Ras-GTP is increased 50% by stimulation of the cells with HGF. HGF-induced motility can be inhibited with microinjections of Rho and dominant negative Rac. HGF stimulation leads to phosphorylation of cytoskeletal proteins such as paxillin and p125FAK. Activation of c-Met by its ligand has also been linked to various signal transduction pathways.

Using the *TPR-MET* oncogene with constitutively activated Met tyrosine kinase activity, we developed a cell line model system suitable to identify small molecule drugs as inhibitors of Met kinase activity. We have developed a small molecule drug specifically inhibiting the Met tyrosine kinase *in vitro* and in cell lines. Inhibition of the Met kinase activity by the novel small molecule drug SU11274 led to time- and dose-dependent reduced cell growth and did not inhibit BCR-ABL, TEL-JAK2, TELPDGF_R, or TEL-ABL tyrosine kinases. SU11274 inhibition of TPRMET kinase activity induced G1 cell cycle arrest and apoptosis with increased Annexin V staining and caspase 3 activity. Also, SU11274 inhibited phosphorylation of key regulators of the PI3K pathway, including AKT, FKHR, or GSK3_. Lung cancer cell lines overexpressing c-Met, H69, and H345 also had growth inhibition with SU11274, with biochemical evidence of inhibition of phosphorylated c-Met. The characterization of SU11274 as an effective inhibitor of Met tyrosine kinase activity illustrates the potential of targeting Met in cancers associated with activated forms of the Met RTK[82].

## Insulin-Like Growth Factor Receptors (IGFRs)

The IGF system is complex and includes several different components. The first is the two growth factors and includes IGF-I and IGF-II, second is the two receptors and includes IGF-IR and IGF-IIR, third is the six high-affinity binding proteins IGFBP-1 through IGFBP-6, and last is the IGFBP proteases. All these parts act in concert to regulate several cellular pathways in a wide variety of tissues throughout the body.

IGF-IR and IGF-IIR are receptor located at the cellular membrane. Both are glycoproteins, but the similarities end there. IGF-IR is an RTK in a family of RTKs that includes the insulin receptor (IR). IGF-IR is upregulated by a variety of growth factors (bFGF, PDGF, EGF), steroids, and hormones (TSH, GH, FSH, LH), while the number of receptors can be decreased by the products of several tumor suppressor genes (WT-1, p53, BRCA-1) as well as IGF-I. IGF-IR is also expressed on almost every cell[83].

Activation of the IGF-I receptor begins with binding of ligand. This induces autophosphorylation and subsequent association with several other proteins leading to downstream signaling. Pathways include those associated with the MAPK which leads to cell growth and proliferation, PI3-K which is associated with prevention of differentiation and apoptosis, JAK (janus kinase)/STAT (signal transducer and activator of transcription), and SOCS (suppressor of cytokine signaling)[84]. This receptor is also closely associated with integrins as well as focal adhesion kinase (FAK) and β-catenin which may be important in apoptosis[85].

IGF-IIR is different from IGF-IR in that this receptor has no intrinsic tyrosine kinase activity, as well as having a higher affinity for IGF-II vs. IGF-I. This receptor may have antiproliferative and proapoptotic activity when binding to IGF-II. This interaction may lead to internalization and degradation of the receptor and ligand[86]. IGF-IIR may also exert its effect by activating TGF-β, or binding to retinoic acid[87].

Within the IGF system, there are two growth factors: IGF-I and IGF-II. Both are single-chain polypeptides with a 62% homology to proinsulin. IGF-I, located on chromosome 12q22, has two promoters which leads to alternative splicing and four precursor proteins, but only one final molecule[88]. This molecule can be synthesized by any tissue in the body, but the liver is the predominant site of synthesis. IGF-I levels are affected by different factors. Growth hormone (GH) is the major regulator of IGF-I production, and IGF-I can subsequently negatively feedback on GH production[89]. IGF-I production can also be affected by other hormones including sex and adrenal steroids, parathyroid hormone, and pituitary hormones (FSH, LH, ACTH, thyrotropin)[83]. IGF-I can also be upregulated by other growth factors such as PDGF, EGF, and bFGF, while fasting and cytokines can downregulate synthesis of IGF-I. Cell proliferation and apoptosis seem to be the major effects of this growth factor.

IGF-II has four promoters and results in various molecular sizes. This gene exhibits genomic imprinting as well as not being regulated by GH. In adults, IGF-II levels are higher than IGF-I levels. Although there are higher levels of IGF-II, its role is most effective during fetal life. In the postnatal period, its role is overtaken by IGF-I. IGF-II also has proliferative and apoptotic effects on cells with which it interacts[83].

The IGFBP family includes six members, with another nine proteins termed insulin-like growth factor binding protein-related proteins (IGFBP-rPs). These proteins are the major determinants of IGF bioavailability. They act to slowly release IGF into the circulation. IGFBPs can prolong IGF's serum half-life, prevent cell growth overstimulation or excessive apoptosis, regulate transport of IGFs to all spaces within the body, and regulate the interaction between IGF and IGFR[90,91]. IGFBPs-1–5 have a high affinity for IGF-I and share a structural similarity.

Gene transcription is regulated by several different hormones, growth factors, and tumor suppressor genes[83]. Each individual IGFBP has a different promoter making them more susceptible to different regulators. Also different tissues produce different IGFBPs. IGFBP-1 is produced primarily by liver and endometrium; IGFBP-2 is produced by neural tissues and prostate and has higher levels during fetal life. IGFBP-3 is also made predominately by the liver, while IGFBP-4 and -5 are synthesized mostly by bone and kidneys. IGFBP-6 is widely produced throughout the body.

The last part of the IGF family includes the IGFBP proteases. These are a heterogeneous family of proteins that include kallikrein-like serine proteases, cathepsins, and matrix metalloproteinases. These molecules degrade IGFBPs and thus alter their interaction with IGFs, which can lead to elevated levels of IGFs and subsequent mitogenic activity of these hormones.

The IGF system has been studied in human malignancies with breast, colon, and prostate cancers being extensively studied[88]. In breast cancer, IGF-I is not produced by breast cancer cells, but is produced by surrounding stromal cells[92]. Thus IGF-I can enhance the mitogenicity of breast cancer cells in both an endocrine and paracrine fashion leading to enhanced breast cancer cell proliferation[93]. It has also been shown that estrogens synergize with IGF-I. Although IGF-II is expressed by both cancer and stromal cells, no evidence has shown there to be an effect on breast cancer cells activity.

Also, IGF-IRs and IRs are more frequently expressed on cancer vs. normal cells. These receptors form heterodimers after binding to IGF-I. This may lead to enhanced metastasis as IGF-IR activation leads to interaction with integrins. IGF-IR is more frequently expressed on tumors that are estrogen receptor (ER) positive[94]. IGF-IIR has no role in cancer cells[95]. IGFBPs also play a role in breast cancer. IGFBP-4 and -5 may prevent apoptosis in cancer cells, while IGFBP-3 inhibits cell growth both directly and by binding IGF-I, making it less readily available to bind the IGF-IR.

Prostate cancer also shows abnormalities in the IGF system. IGF-I is overexpressed by stromal cells, but not by cancer cells and levels directly correlate with cancer risk. As with breast cancer, IGF-II is produced by both stromal and cancer cells, but no effect has been observed[96]. IGF-IR is usually detected only in early cancer. IGFBPs levels may be increased, decreased, or unchanged and IGFBP-3 has been shown to be related to decreased cancer risk. Androgen receptors (ARs) expression is induced by IGFs and can enhance IGF-IR signaling. ARs may also have an effect on IGFBPs production.

In colon cancer, IGF-I acts as an antiapoptotic signal and can upregulate VEGF[97,98]. There is also a direct correlation between IGF-I levels and cancer risk. IGF-II expression is also upregulated in cancer cells. IGF-IR directly correlates with grade and stage and can be overexpressed in cancer cells[99]. IGFBPs have different effects in these cancer cells.

In lung cancer, many studies have shown that IGF receptors are expressed in lung tumor tissues[100,101,102]. IGF-II overexpression has been shown to be sufficient to induce lung tumorigenesis in an *in vivo* mouse model. While IGF-II levels are associated with a poor prognosis in pulmonary adenocarcinoma, prospective studies have not been able to demonstrate a link between IGF-I or IGF-II levels, and lung cancer risk[103]. However, high tumor IGF-II immunoreactivity has been correlated with decreased survival in patients with lung adenocarcinoma, suggesting that tissue-specific levels of IGF-II

may be more relevant than plasma levels. Overall, more extensive studies are required to establish the precise role of IGF in lung cancer[104].

The IGF system has been implicated in the pathogenesis as well as metastasis of malignancies. Agents that alter the functioning of this system have been proposed and experimented[105], but yet have not shown promise. Agents such as somatastatin, which is known to downregulate the GH/IGF-I axis, has been proposed and used in patients with metastatic disease; results are disappointing. Agents that upregulate IGFBP-3 have shown antitumor effects *in vitro* and *in vivo*[106]. No clinical trials have yet begun for these inhibitors.

## Eph Receptors

The Eph receptor family is the largest family of RTKs. Ephrins, the Eph receptor ligands, are molecules located on cell surfaces as well. Interaction between RTK and ligand occurs only in areas of direct cell-cell contact[107,108]. Within this family, there is subdivision into two subclasses: A and B. There are nine different EphA RTKs (EphA1–EphA9) that bind to and are activated by six A-ephrins (ephrinA1–ephrinA6). There are six EphB RTKs (EphB1–EphB6) that interact with three B-ephrins (ephrinB1–ephrinB3). A-type receptors bind all or most A-type ligands, while B-type receptors bind to B-type ligands with the exception of EphA4, which can bind both A-type and most B-type ligands. This family of receptors and ligands send messages bidirectionally, which sets them apart from the other RTKs that send messages unidirectionally.

Eph receptors are type-I transmembrane proteins that have a highly conserved N-terminal domain used for ligand recognition and binding, a cysteine-rich region, and two fibronectin-type III repeats which may be involved receptor dimerization. The ligand-binding motifs are unique to this family of RTKs and share no homology with other ligand-binding domains contained in other RTKs. On the intracellular side of the membrane, there is a juxtamembrane region, a conserved kinase domain, a sterile-α-motif (SAM) domain, and a PSD95/Dlg/ZO1 (PDZ)-binding motif[108,109,110].

Ephrins are membrane-bound molecules that possess a unique N-terminal receptor binding domain (RBD) that is linked to the cell membrane by a 40 amino acid polypeptide. A-ephrins are linked to the cell via a glycosylated phosphatidylinositol (GPI) linkage. B-ephrins are transmembrane proteins that contain a highly conserved intracellular domain, which contains a C-terminal PDZ-binding motif. The ephrin sequences are unique to this family and have no similarities with any other proteins[107].

The family members of this RTK family have been reported in numerous cancers and cancer cell lines. EphA1-A3, EphB1-B6, ephrinA1, and ephrinB1-B3 have been identified in lung cancers as well as lung cancer cell lines[111]. EphA1 and EphA3 are noted to be upregulated. Ephs and ephrins regulate cell migration, repulsion, vascular development, axonal guidance, and adhesion or attachment to extracellular matrix. The role of Eph receptors and ephrins in carcinogenesis is less clear. The major role of Eph receptors and ephrin ligands may be angiogenesis or metastasis and not in transformation of normal cells to cancer cells.

After binding between Eph RTK and ephrin occurs, high-affinity heterodimers are created. Heterodimers can then pair, creating larger tetramers with ligand on one side and RTK on the other. It is the tetramerization that allows for the bidirectional signaling. There are many different pathways that are activated by this family of RTKs. One pathway includes the Rho family of GTPases, and includes Rho, Rac, and Cdc42, which can exert an effect on the cellular cytoskeleton[112]. The link between Eph receptors and GTPases is via Ephexin (Eph-interacting exchange protein), which is a novel guanine nucleotide exchange factor for Rho GTPases[113]. With activation of Ephexin, RhoA is activated while Rac1 and Cdc42 are inhibited. This leads to increased contraction of the cytoskeleton.

Another pathway that is affected by Eph receptors is the ERK/MAPK (extracellular-signal-regulated kinase/mitogen-activated protein kinase) pathway. Miao et al.[114] demonstrated that ephrinA1 stimulation led to suppression of the ERK/MAPK pathway leading to inhibition of proliferation of immortalized prostatic epithelial cells and primary prostatic cancer cells. It was also shown that there was reduced activity in the EGF- or PDGF-simulated ERK/MAPK pathway after stimulation of ephrinA1. Similar findings were demonstrated with stimulation of EphB2 receptors, leading to growth-cone collapse and neurite contraction. Inhibition of the ERK/MAPK pathway leads to the reduction of mitogenic signals and induces cytoskeletal changes.

The Eph-ephrin family also interacts with numerous cellular proteins. Stimulation of EphB2 receptors leads to phosphorylation of R-Ras, which leads to decreased integrin support[115]. Activation of EphA2 leads to decreased integrin-mediated adhesion by inducing dephosphorylation of FAK[116]. This leads to reduced cellular adhesion. Activation of EphB1 receptors leads to increased integrin-mediated cell attachment[117]. This process is via numerous intermediaries including low-molecular-weight protein tyrosine-phospatase (LMW-PTP), Nck, and Nck-interacting kinase (NIK). Increased integrin-mediated cell adhesion is also regulated by the EphA8 receptor via the p110γ regulatory subunit of PI3-K.

In this family of RTKs, ephrins are also responsible for signaling within their own cells after activation by binding to Eph receptors. Ephrin B ligands interact with the adaptor protein Grb4[118]. The SH2 domain of this protein, binds to ephrinB ligands after they are phosphorylated, which can occur after activation of other RTKs such as PDGF. Cowan and Henkemeyer[119] showed that recruitment of Grb4 to ephrinB1 led to loss of polymerized F-actin structures leading to the disassembly of focal adhesions.

EphrinB ligands can also interact with other molecules via its PDZ-binding motif. The adaptor molecules also contain a PDZ domain and include glutamate-receptor-interacting-protein-1 (GRIP1), GRIP2, syntenin, protein kinase C-interacting protein Pick 1, and PTP-BL phosphotase[120,121,122,123].

In studies pertinent to lung cancer, differential gene expression in normal lung vs. tumor tissue showed that Ephrin A3 was upregulated 26 times in the tumor[124]. Ephrin A2 was present only in the tumor and not detected in normal lung. Significant differences were also observed in tumors of other organs. Another study showed that levels of EphA2 expression in NSCLC can provide information about clinical outcome[125]. The highest levels of EphA2 expression were found in those patients who went on to develop brain metastases, whereas low levels were expressed in patients who did not relapse or who developed contralateral disease.

These findings show the clinical importance of further study into these receptors. Detection of circulating EphA2 levels could serve as a diagnostic marker for metastatic disease, as well as serve as a guide to clinical management of metastatic disease. Also, the absence of EphA2 in normal lung tissue makes it an ideal receptor for antibody targeting, which can specifically target tumor tissue, while minimizing adverse effects to normal cells[126]. Such antibodies, while being used for *in vitro* studies, have not been available for the purpose of treating patients with cancer.

The role of the Eph family is diverse and remains unclear, and warrants further study. Different receptors and ligands serve very different purposes in a wide variety of tissues.

## CONCLUSION

Several different RTKs have been shown to play a role in carcinogenesis of several different malignancies including lung cancers. RTKs are important in making tumor cells immortal and enhancing their ability to metastasize. It is with better understanding of how these molecules work and activate cellular pathways that better treatments can be developed. Both monoclonal antibodies and tyrosine kinase inhibitors have been developed, but in majority are still in the infancy of clinical trials. More human trials need to be conducted with these newer agents alone or in combination with existing chemotherapies/radiation therapy/surgery to determine which ones will be effective in treating those with lung cancer and other malignancies.

## ACKNOWLEDGMENTS

R. Salgia is funded by a Research Scholar Award from the American Cancer Society (RSG CCE-102606), NIH/NCI (RO1, CA 100750-01), and the Verto Institute.
